# Supplementary material for: Cerebral Protection in TAVR—Can We Do Without? A Real-World All-Comer Intention-to-Treat Study—Impact on Stroke Rate, Length of Hospital Stay, and Twelve-Month Mortality
Source: J Pers Med. 2022 Feb 21;12(2):320. doi: 10.3390/jpm12020320 (PMC8878932; doi:10.3390/jpm12020320)
Supplement: Supplementary file 1 [file jpm-12-00320-s001.zip › Supplemental Material.pdf]

Supplemental Table S1. Propensity-score matched analysis - Cerebrovascular events at 72 hours

Propensity-score matched analysis		
	**Odds ratio (95%CI)**	**p-Value**
Baseline characteristics		
Sex, female	0.828 (0.272-2.519)	0.739
Age	1.012 (0.945-1.085)	0.730
Body mass index	1.007 (0.916-1.107)	0.539
Arterial hypertension	0.671 (0.188-2.392)	0.552
Diabetes mellitus	1.323 (0.526-3.325)	0.552
Peripheral artery disease	0.386 (0.050-2.963)	0.360
Coronary artery disease	1.885 (0.663-5.359)	0.235
Carotid stenosis >70%	2.164 (0.747-6.268)	0.155
Hyperlipidemia	1.470 (0.480-4.505)	0.500
Previous stroke	-	
Atrial fibrillation	1.108 (0.401-3.060)	0.844
COPD	1.510 (0.421-5.414)	0.527
Previous cardiac surgery	1.495 (0.478-4.676)	0.489
Previous syncope	1.570 (0.495-4.981)	0.444
NYHA functional class	0.945 (0.520-1.716)	0.853
CCS≥III	1.172 (0.260-5.277)	0.836
NT-proBNP (log.)	0.686 (0.297-1.585)	0.378
Creatinine	0.745 (0.353-1.571)	0.439
Oral anticoagulation	1.965 (0.751-5.141)	0.169
SAPT	1.732 (0.670-4.474)	0.257
DAPT	0.685 (0.221-2.119)	0.511
EuroSCORE II	1.018 (0.955-1.085)	0.593
Echocardiographic parameters		
AV mPG	0.980 (0.947-1.014)	0.240
AV pPG	0.993 (0.971-1.015)	0.519
AVA	1.726 (0.126-23.551)	0.682
AV Vmax	0.787 (0.387-1.598)	0.507
LVEF	0.990 (0.944-1.037)	0.664
Procedural characteristics		
Self-expanding valve	1.373 (0.364-5.188)	0.640
Sentinel^{TM}	0.115 (0.026-0.505)	**0.004**
Valve size	1.081 (0.925-1.264)	0.325
Predilation	0.885 (0.296-2.648)	0.828
Postdilation	1.179 (0.397-3.498)	0.767
Procedure time	1.012 (1.002-1.021)	0.19
Implantation >1 valve	23.609 (4.432-125.763)	**<0.001**
Fluoroscopy time	1.053 (1.012-1.095)	**0.011**

AV indicates aortic valve; AVA, aortic valve area; CCS, Canadian Cardiovascular Society; COPD, chronic obstructive pulmonary disease; DAPT, dual antiplatelet therapy; log, logarithmized; LVEF, left ventricular ejection fraction; mPG, mean pressure gradient; NYHA, New York Heart Association; pPG, peak pressure gradient; NT-proBNP, N-terminal pro brain natriuretic peptide; SAPT, single antiplatelet therapy; Vmax, maximal velocity

Supplemental Table S2 – Propensity-score matched analysis - Six months mortality

Propensity-score matched analysis		
	**Odds ratio (95%CI)**	**p-Value**
Baseline characteristics		
Sex, female	2.698 (1.359-5.356)	**0.005**
Age	0.996 (0.955-1.038)	0.836
Body mass index	0.987 (0.930-1.048)	0.672
Arterial hypertension	0.534 (0.260-1.098)	0.088
Diabetes mellitus	1.582 (0.908-2.755)	0.105
Peripheral artery disease	2.323 (1.190-4.531)	**0.013**
Coronary artery disease	1.485 (0.797-2.769)	0.213
Carotid stenosis >70%	1.687 (0.844-3.372)	0.139
Hyperlipidemia	0.789 (0.436-1.429)	0.435
Previous stroke	0.761 (0.237-2.444)	0.646
Atrial fibrillation	2.525 (1.355-4.706)	**0.004**
COPD	1.031 (0.438-2.426)	0.943
Previous cardiac surgery	2.016 (1.049-3.873)	**0.035**
Previous syncope	1.152 (0.557-2.379)	0.703
NYHA functional class	1.886 (1.183-3.007)	**0.008**
CCS≥III	0.625 (0.195-2.010)	0.431
NT-proBNP (log.)	1.367 (1.095-1.707)	**0.006**
Creatinine	1.169 (0.941-1.451)	0.159
Oral anticoagulation	1.530 (0.842-2.781)	0.163
SAPT	1.214 (0.648-2.275)	0.545
DAPT	0.882 (0.444-1.749)	0.718
EuroSCORE II	1.056 (1.026-1.087)	**<0.001**
Echocardiographic parameters		
AV mPG	0.978 (0.958-0.998)	**0.031**
AV pPG	0.983 (0.969-0.997)	**0.018**
AVA	1.844 (0.360-9.438)	0.462
AV Vmax	0.602 (0.390-0.930)	**0.022**
LVEF	0.980 (0.952-1.008)	0.167
Procedural characteristics		
Self-expanding valve	0.906 (0.394-2.083)	0.817
Sentinel^{TM}	0.484 (0.265-0.884)	**0.018**
Valve size	1.064 (0.969-1.169)	0.195
Predilation	0.735 (0.390-1.384)	0.340
Postdilation	0.773 (0.390-1.530)	0.459
Procedure time	1.014 (1.008-1.021)	**<0.001**
Implantation >1 valve	2.781 (0.676-11.441)	0.156
Fluoroscopy time	1.026 (0.999-1.053)	0.063

AV indicates aortic valve; AVA, aortic valve area; CCS, Canadian Cardiovascular Society; COPD, chronic obstructive pulmonary disease; DAPT, dual antiplatelet therapy; log, logarithmized; LVEF, left ventricular ejection fraction; mPG, mean pressure gradient; NYHA, New York Heart Association; pPG, peak pressure gradient; NT-proBNP, N-terminal pro brain natriuretic peptide; SAPT, single antiplatelet therapy; Vmax, maximal velocity

Supplemental Table S3 – Propensity-score matched analysis - Length of hospital stay

Propensity-score matched analysis		
	**Regression coefficient (95%CI)**	**p-Value**
Baseline characteristics		
Sex, female	0.478 (-1.425, 2.380)	0.622
Age	0.134 (0.017, 0.251)	**0.024**
Body mass index	-0.085 (-0.236, 0.066)	0.271
Arterial hypertension	-0.699 (-3.224, 1.826)	0.587
Diabetes mellitus	0.542 (-1.125, 2.209)	0.523
Peripheral artery disease	0.821 (-1.662, 3.304)	0.516
Coronary artery disease	1.940 (0.297, 3.583)	**0.021**
Carotid stenosis >70%	-0.535 (-2.841, 1.772)	0.649
Hyperlipidemia	-0.509 (-2.287, 1.269)	0.574
Previous stroke	0.008 (-2.998, 3.015)	0.996
Atrial fibrillation	0.805 (-0.969, 2.578)	0.373
COPD	-0.453 (-2.934, 2.028)	0.720
Previous cardiac surgery	-0.280 (-2.529, 1.968)	0.807
Previous syncope	2.247 (0.144, 4.349)	**0.036**
NYHA functional class	0.758 (-0.246, 1.761)	0.138
CCS≥III	-0.309 (-3.078, 2.459)	0.826
NT-proBNP (log.)	2.326 (1.030, 3.622)	**<0.001**
Creatinine	0.643 (-0.216, 1.502)	0.142
Oral anticoagulation	0.177 (-1.568, 1.921)	0.842
SAPT	0.470 (-1.308, 2.249)	0.603
DAPT	-0.048 (-1.876, 1.780)	0.959
EuroSCORE II	0.117 (-0.013, 0.247)	0.078
Echocardiographic parameters		
AV mPG	-0.030 (-0.084, 0.024)	0.275
AV pPG	-0.003 (-0.040, 0.034)	0.871
AVA	-4.489 (-10.387, -0.791)	**0.023**
AV Vmax	0.085 (-1.188, 1.358)	0.896
LVEF	-0.101 (-0.192, -0.009)	**0.031**
Procedural characteristics		
Self-expanding valve	2.665 (0.345, 4.984)	**0.024**
Sentinel^{TM}	-2.005 (-3.561, -0.450)	**0.012**
Valve size	-0.027 (-0.317, 0.263)	0.856
Predilation	-1.022 (-2.866, 0.823)	0.277
Postdilation	-1.063 (-2.939, 0.830)	0.263
Procedure time	0.024 (-0.002, 0.050)	0.073
Implantation >1 valve	3.382 (-2.644, 9.408)	0.271
Fluoroscopy time	0.128 (0.037, 0.219)	**0.006**

AV indicates aortic valve; AVA, aortic valve area; CCS, Canadian Cardiovascular Society; COPD, chronic obstructive pulmonary disease; DAPT, dual antiplatelet therapy; log, logarithmized; LVEF, left ventricular ejection fraction; mPG, mean pressure gradient; NYHA, New York Heart Association; pPG, peak pressure gradient; NT-proBNP, N-terminal pro brain natriuretic peptide; SAPT, single antiplatelet therapy; Vmax, maximal velocity
